# Non-targeted metabolomics analysis reveals the mechanism of arbuscular mycorrhizal symbiosis regulating the cold-resistance of *Elymus nutans*

**DOI:** 10.3389/fmicb.2023.1134585

**Published:** 2023-08-07

**Authors:** Haijuan Zhang, Hexing Qi, Guangxin Lu, Xueli Zhou, Junbang Wang, Jingjing Li, Kaifu Zheng, Yuejun Fan, Huakun Zhou, Jiuluan Wang, Chu Wu

**Affiliations:** ^1^College of Agriculture and Animal Husbandry, Qinghai University, Xining, China; ^2^Experimental Station of Grassland Improvement of Qinghai Province, Xining, China; ^3^National Ecosystem Science Data Center, Key Laboratory of Ecosystem Network Observation and Modeling, Institute of Geographic Sciences and Natural Resources Research, Chinese Academy of Sciences, Beijing, China; ^4^College of Agriculture and Animal Husbandry Science and Technology Vocational of Qinghai Province, Xining, China; ^5^Qinghai Provincial Key Laboratory of Restoration Ecology of Cold Area, Northwest Institute of Plateau Biology, Chinese Academy of Sciences, Xining, China; ^6^Grassland Station of Qinghai Province, Xining, China; ^7^College of Horticulture and Gardening, Yangtze University, Jingzhou, Hubei, China

**Keywords:** arbuscular mycorrhizal fungi, Gramineae grasses, stress resistance, resistance mechanism, metabolomics

## Abstract

*Elymus nutans* is a perennial grass of the Gramineae family. Due to its cold-resistance and nutrition deficiency tolerance, it has been applied to the ecological restoration of degraded alpine grassland on the Qinghai-Tibet Plateau. As an important symbiotic microorganism, arbuscular mycorrhizal fungi (AMF) have been proven to have great potential in promoting the growth and stress resistance of Gramineae grasses. However, the response mechanism of the AMF needs to be clarified. Therefore, in this study, *Rhizophagus irregularis* was used to explore the mechanism regulating cold resistance of *E. nutans*. Based on pot experiments and metabolomics, the effects of *R. irregularis* were investigated on the activities of antioxidant enzyme and metabolites in the roots of *E. nutans* under cold stress (15/10°C, 16/8 h, day/night). The results showed that lipids and lipid molecules are the highest proportion of metabolites, accounting for 14.26% of the total metabolites. The inoculation with *R. irregularis* had no significant effects on the activities of antioxidant enzyme in the roots of *E. nutans* at room temperature. However, it can significantly change the levels of some lipids and other metabolites in the roots. Under cold stress, the antioxidant enzyme activities and the levels of some metabolites in the roots of *E. nutans* were significantly changed. Meanwhile, most of these metabolites were enriched in the pathways related to plant metabolism. According to the correlation analysis, the activities of antioxidant enzyme were closely related to the levels of some metabolites, such as flavonoids and lipids. In conclusion, AMF may regulate the cold-resistance of Gramineae grasses by affecting plant metabolism, antioxidant enzyme activities and antioxidant-related metabolites like flavonoids and lipids. These results can provide some basis for studying the molecular mechanism of AMF regulating stress resistance of Gramineae grasses.

## Introduction

Alpine grassland is the most important ecosystem of the Qinghai-Tibet Plateau, accounting for more than half of the plateau area (Li et al., 2018a,b). It has ecological functions such as supporting the development of grassland husbandry, regulating climate, and maintaining species diversity (Shang et al., 2014; Sun et al., 2018). However, the alpine grassland has been seriously degraded because of global climate change (Yang et al., 2017), overgrazing, engineering activities such as road building, herbal medicine excavation, and the infestation of rodents and pests, resulting in the abnormal functioning of the ecosystem (Bao et al., 2015; Liu et al., 2022a,b). Fencing, rotation grazing, supplementary seeding of fine herbages, and reasonable fertilization have been proven effective restoration strategies (Li et al., 2018a,b, 2023). However, under the adapted management of degraded alpine grassland, the combination of herbage replanting and microbial controlling is crucial to restoring the ecological functions of the degraded alpine grassland (Zhou et al., 2020).

Gramineae grasses are the main seeding plants applied in the restoration of degraded alpine grassland (Tian et al., 2021). As a perennial Gramineae grass, *Elymus nutans* was applied in the re-establishment of artificial grassland, natural grassland reseeding, and ecological restoration of the degraded grassland in the alpine region of the Qinghai-Tibet Plateau because of its characteristics of high yield and quality, cold resistance, and nutrition deficiency tolerance. However, the restrictions of provenance and extreme climatic conditions lead to the highly unstable growth of grasses, which is prone to secondary degradation (Song and Yu, 2015). At the same time, the resource waste and the substantial production costs also bring difficulties to grass-based livestock development husbandry and regional ecological restoration.

As an important symbiotic microorganism that widely exists in soil, arbuscular mycorrhizal fungi (AMF) can form mycorrhizal structures such as mycelia, vesicles, spores, and arbuscles with more than 80% of terrestrial higher plants, including Gramineae (Salvioli et al., 2016; Genre et al., 2020). Then, it can promote plant growth, such as increasing plant height and biomass accumulation, expanding the contact area of plant roots, etc. (van der Heijden et al., 2015; Gao et al., 2020; Li et al., 2022). Meanwhile, this mutuality can also enhance the resistance of plants to environmental stresses, such as low temperature (Liu et al., 2013), drought (Ruiz-Lozano et al., 2016; Chandrasekaran, 2022), salt (Aroca et al., 2013; Duc et al., 2021; Zai et al., 2021), and heavy metals (Dhalaria et al., 2020; Riaz et al., 2021). Therefore, it has been applied to improve the resistance of plants and repair soil with heavy metal pollution. However, only a few studies explored the molecular mechanism involved in plant resistance to environmental stresses under symbiosis. These researches indicated that AMF improved plant resistance to low-temperature by increasing the contents of secondary metabolites, including phenols, flavonoids, lignin, DPPH activity, and phenolic compounds (Chen et al., 2013), and accumulating content of proline via enhancement of the Glu and Orn synthetic pathways (Liu et al., 2022a,b); enhancing drought tolerance by altering compositions of fatty acid and levels of saturation (Wu et al., 2019), and declining almost all differential terpenoids (Liang et al., 2021). Meanwhile, sugars and lipids were positively modulated (Bernardo et al., 2019); Under salt stress, activities of catalase and peroxidase, contents of proline and phenolic were increased to improve salt tolerance (Duc et al., 2021; Israel et al., 2022). Studies have also shown that the effects of AMF on plant stresses resistance were also closely related to metabolic pathways related to organic acid and amino acid (Liu et al., 2023) and secondary metabolites, such as phytohormones and signaling molecules (Bahadur et al., 2019). However, these pieces of evidence may be insufficient to explain how AMF affects the stress resistance of Gramineae grasses, especially in the alpine regions.

Therefore, based on our previous experimental results and the geographical distribution characteristics of AMF on the Qinghai-Tibet Plateau, in this study, *E. nutans* and *Rhizophagus irregularis* were used to investigate the mechanism that AMF inoculation increased cold-resistance of Gramineae grasses. Through pot experiments and UHPLC-MS/MS-based metabonomics, we studied the effects of AMF inoculation on the antioxidant enzyme activities and metabolite levels of *E. nutans* roots under cold stress (15/10°C, 16/8 h, day/night). This study aimed to explore the possible molecular mechanism of AMF inoculation regulating the cold-resistance of Gramineae grasses.

## Materials and methods

### Sources of experimental materials

The seeds of *E. nutans* ‘Aba’ were provided by the Experimental Station of Grassland Improvement of Qinghai Province, which were purchased from Sichuan Chuancao Ecological Grassland Technology Development Co., Ltd., with a germination rate ≥95%. Before sowing, the seeds were sterilized with 10% H_2_O_2_ for 10 min, and then washed with sterile water 5 times, finally dried with filter paper. *R. irregularis* was donated by Professor Wu Chu at Yangtze University and proliferated by symbiosis with *Trifolium repens*. During proliferation, the cultivation substrate was the soil collected from the Experimental Station of Grassland Improvement of Qinghai Province, with a total nitrogen level of 1469.56 mg/kg, ammonia nitrogen of 21.89 mg/kg, nitrate nitrogen of 64.98 mg/kg, and organic matter of 4.07%. The substrate was dried, passed through a 2 mm soil sieve, and sterilized at 121°C for 2 h before cultivation. Plastic pots (18 cm × 15 cm × 13 cm) were sterilized with 75% alcohol and used for the experiment.

### Research design

In this experiment, the pot culture method was applied. First, two treatments were set up, one was the group inoculated with *R. irregularis*, and the other was not inoculated. Next, each treatment was repeated 10 times, 20 pots in total. The specific steps: first, 400 g of the sterilized cultivation substance in a sterilized pot was evenly covered with 20 g of the AMF inoculum mentioned above, and then evenly covered with 60 g of the sterilized cultivation substance, and 100 mL of sterile water was sprayed. The grass seeds were sowed, 50 grains per pot, and 40 g of the cultivation substance was used to cover these seeds. Finally, a little sterile water was sprayed. After 7 days of germination, the seedlings were thinned, leaving 30 seedlings per pot. All the seedlings were continuously cultivated in a greenhouse with natural light, 22°C day/12°C night. The seedlings were cultivated for 60 days, and the Hoagland nutrient solution (Waheed et al., 2019) was provided once a week, 100 mL per pot.

After 60 days of cultivation, the 10 potted plants without AMF inoculation were randomly divided into two groups, i.e., normal temperature (NT) and low temperature (LT), 5 pots in each group. The 10 potted plants inoculated with *R. irregularis* were also randomly divided into two groups, i.e., NT-AMF and LT-AMF, 5 pots in each group. The two treatment groups, i.e., LT and LT-AMF, were cultivated in an RDN-type artificial climate chamber (15°C day/10°C night, 16 h light/8 h dark) for 10 days, while the other two treatment groups, i.e., NT and NT-AMF, were cultivated in an RDN-type artificial climate chamber (22°C day/12°C night, 16 h light/8 h dark) for 10 days. For all the four treatment groups, 60% relative humidity and 3,000 lx light density were provided in the climate chambers. After cultivation of 10 days, three complete plant roots were randomly dug from the five biological replicates of each group and rinsed, and then these roots were cut into 1–2 cm root segments. Finally, the mycorrhizal infection was detected by fixing these root segments in the formaldehyde-acetic-acid (FAA) solution and storing under 4°C. At the same time, 3 complete plant roots were randomly collected from 5 biological replicates of each group and mixed into one sample, 9 replicates, thus 9 samples in total. Out of them, 6 samples were used for UHPLC-MS/MS non-targeted metabonomic analysis, and 3 samples were used to determine antioxidant enzyme activities. The samples used for metabonomic analysis were cleaned with the 1 × PBS and treated in liquid nitrogen for 15 min, and subsequently were stored under −80°C. The samples used to determine the activities of antioxidant enzyme were rinsed with sterilized water and treated in liquid nitrogen for 15 min and subsequently stored under −80°C.

### Mycorrhizal infection detection

The mycorrhizal infection rate in the roots of *E. nutans* was determined using the method by Blazkova et al. (2021), and trypan blue staining method was used to detect mycorrhizal structure including vesicles and hyphaes (Jill et al., 2020).

### Assay of antioxidant enzyme activities

The activities of ascorbate peroxidase (APX), peroxidase (POD), superoxide dismutase (SOD), and catalase (CAT) were determined according to the methods of Jin et al. (2023) and Zhang et al. (2023).

### Metabolite extraction and UHPLC-MS/MS analysis

At first, 100 mg of liquid nitrogen-ground root sample was placed in an Eppendorf tube, and 500 μL of 80% methanol aqueous solution (Thermo Fisher, United States) was added. Next, after vortex oscillation until mixed evenly, ice bath for 5 min, centrifugation for 20 min (15,000 g, 4°C, Scilogex, United States), 400 μL of supernatant with mass spectrometry grade water (Merck, Germany) was diluted until the methanol concentration was 53%. The solution was centrifuged at 15000 *g* and 4°C for 20 min to collect the supernatant, and the sample was injected for UHPLC-MS/MS analysis (Want et al., 2012; Silva et al., 2021). Equal volume samples from each experimental sample were taken and mixed as QC samples. 53% methanol-water solution was applied to replace the experimental sample, and the pretreatment process was the same as the experimental sample.

UHPLC-MS/MS analysis was performed using a Vanquish UHPLC instrument (Thermo Fisher, Germany). Chromatographic conditions: chromatographic column, Hypesil Gold column (100 mm × 2.1 mm, 1.9 μ m), 40°C; positive ion mobile phase, A-0.1% formic acid, B-methanol; negative ion mobile phase, A-5 mM ammonium acetate (pH 9.0), B-methanol; flow rate, 0.2 mL/min; the chromatographic gradient elution procedure was: 0–1.5 min, 98% solvent A; 1.5–3 min, 15% solvent A; 3–10 min, 0% solvent A; 10–12 min, 98% solvent A; the sample mass spectrum signal under positive and negative ion modes were collected through the Q Exactive^™^ HF-X (Thermo Fisher, Germany). Mass spectrum condition: *m*/*z*, 100–1,500; spray voltage, 3.5 kV; sheath gas flow rate, 35 psi; aux gas flow rate, 10 L/min; capillary temperature, 320°C; S-lens RF level, 60; aux gas heater temperature, 350°C.

### Data analysis

The original data obtained from UHPLC-MS/MS analysis were imported into Compound Discoverer 3.1 (CD 3.1, Thermo Fisher). After screening the retention time, mass charge ratio, and other parameters of each metabolite, the retention time deviation of 0.2 min and the mass deviation of 5 ppm were set to align the peaks of different samples. A series of settings, including the mass deviation of 5 ppm, signal strength deviation of 30%, the signal-to-noise ratio of 3, the minimum signal strength, additive ions, and other information, were set to extract the peaks. The peak area was quantified and the target ions were integrated to predict the molecular formula by molecular ion peak and fragment ions. Compared with the mzCloud, mzVault, and Masslist databases and obtained the identification and relative quantitative of metabolites, and annotated them using KEGG, HMDB and LIPIDMaps databases.

The SIMCA 14.1 was applied for PCA and PLS-DA analysis and random array test (200 times), to know the overall distribution characteristics of the samples and the stability of the entire analysis process. The histograms, classification ring diagram of metabolites, differential metabolite volcano pots and correlation heatmaps were drawn by the Origin 22. The TBtools (Chen et al., 2020) was applied to draw the Venn diagrams and cluster heatmaps. The KEGG enrichment bubble diagrams were drawn on the bioinformatics cloud platform.^1^

## Results

### Mycorrhizal infection

**Figure 1:**
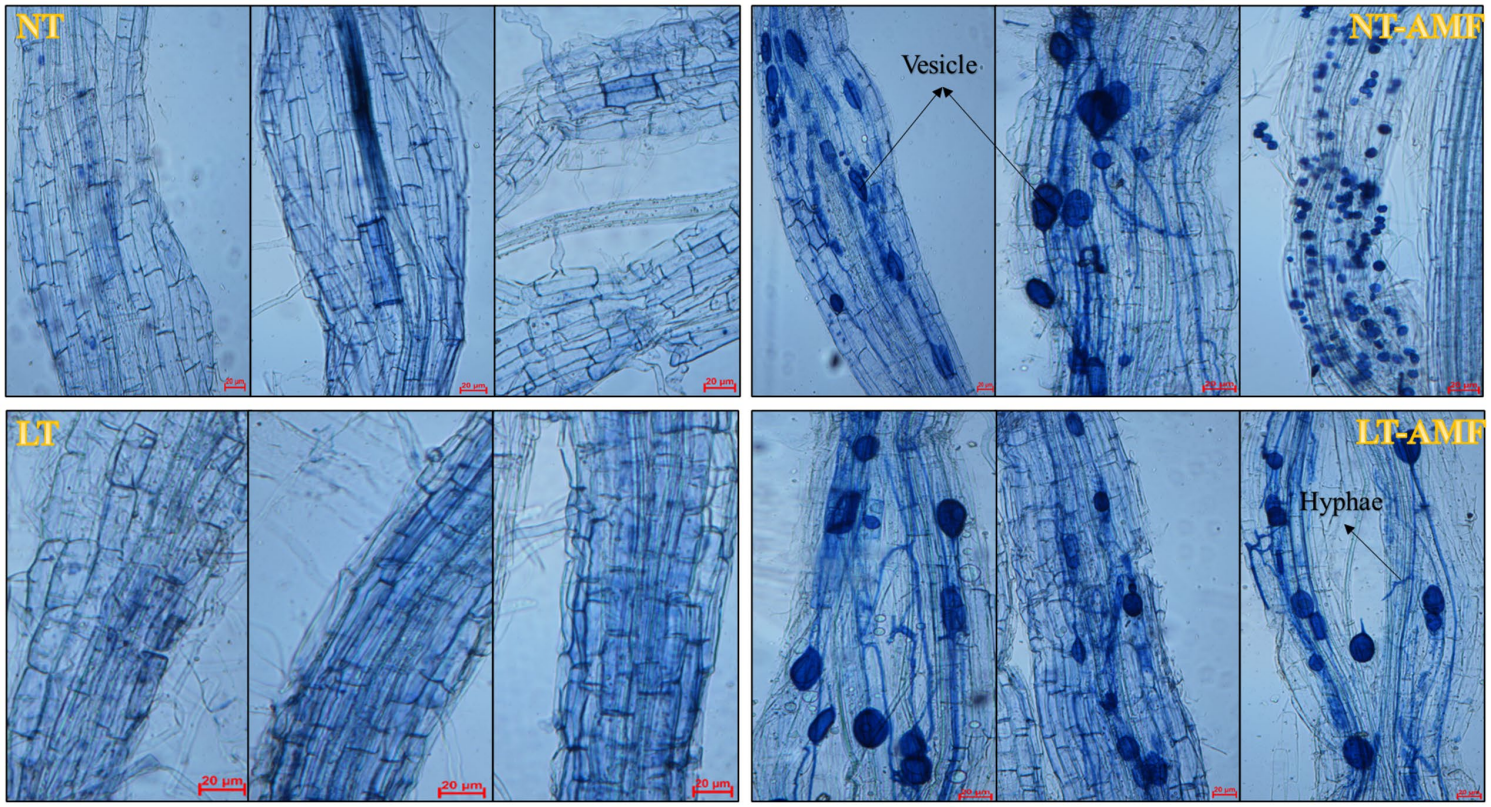
Detection of mycorrhizal infection of root samples. NT, normal temperature; NT-AMF, normal temperature + *Rhizophagus irregularis*; LT, low temperature; LT-AMF, low-temperature + *R. irregularis*. In the figure, the length of scale bars was 20 μm, which was used to measure the size of vesicles and hyphae. The same below.

The mycorrhizal infection of root samples from 4 treatment groups (NT, NT-AMF, LT, LT-AMF) was detected by trypan blue staining, to clear the infection of *R. irregularis* in roots of *E. nutans* ‘Aba’. According to the mycorrhizal infection diagrams of root samples (Figure 1), *R. irregularis* successfully infected the root cortex of *E. nutans* ‘Aba’ and mycorrhizal structures were observed, such as vesicles and hyphae, suggesting that the inoculation experiment was effective for subsequent tests.

### Antioxidant enzyme activities

**Figure 2:**
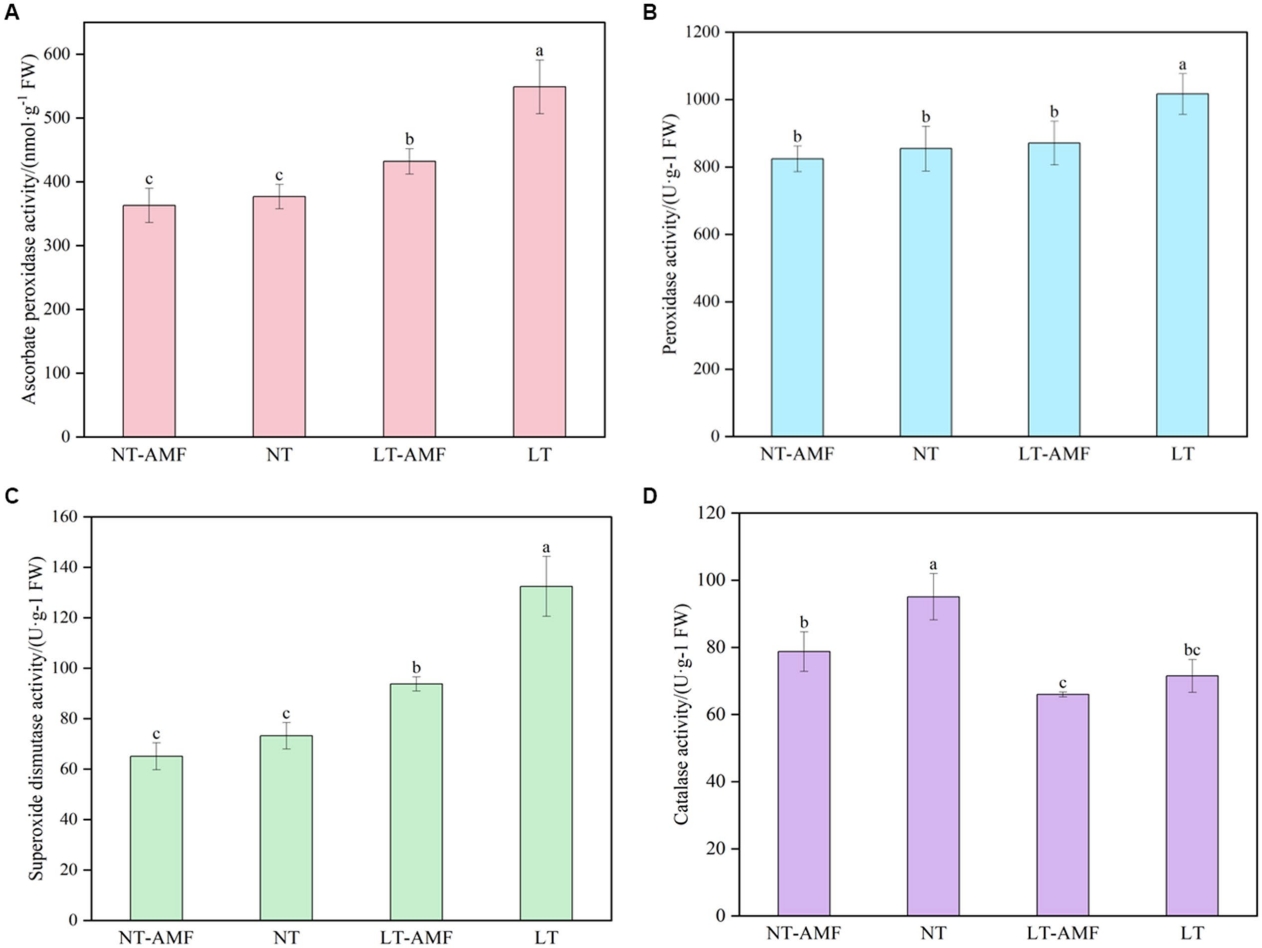
The activity of antioxidant enzymes. **(A)** APX activity, **(B)** POD activity, **(C)** SOD activity, **(D)** CAT activity. In the figure, different lowercase letters indicate significant one-way ANOVA results between groups at *p* = 0.05.

According to the results of the four treatment groups shown in Figure 2, the inoculation of *R. irregularis* at room temperature showed no significant effect on the activities of antioxidant enzyme (i.e., APX, POD, SOD) in the roots of *E. nutans* ‘Aba’ (NT vs. NT-AMF). However, after 10 days of low-temperature treatment at 15°C/10°C (16 h/8 h, day/night), the activities of APX, POD and SOD in the inoculated group (LT-AMF) and the non-inoculated group (LT) increased or significantly increased (*p* < 0.05), while the activity of CAT significantly decreased (*p* < 0.05). The activities of APX, POD and SOD in the non-inoculated group (LT) were significantly higher than those in inoculated group (LT-AMF) (*p* < 0.05), respectively, but there was no significant difference in CAT activity between the two groups.

### Metabolite detection and classification

**Figure 3:**
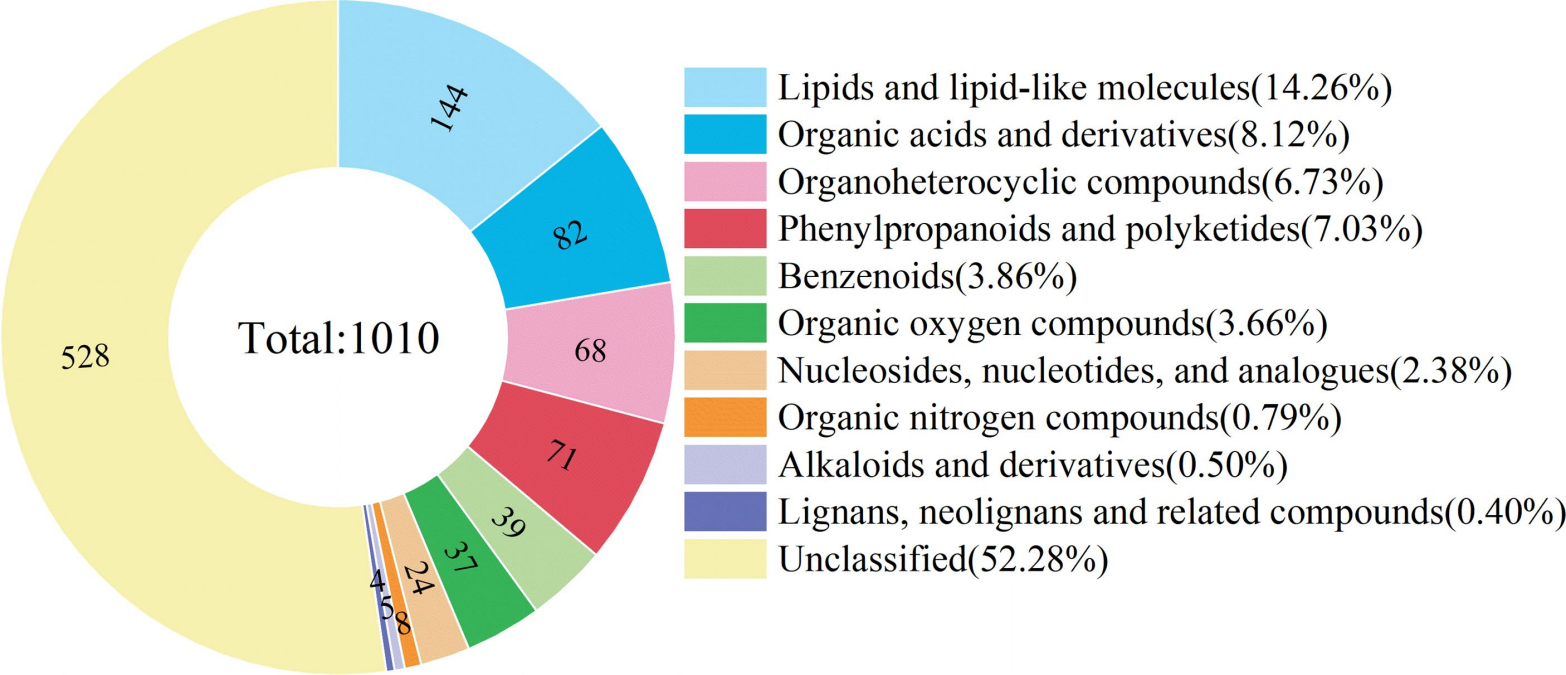
Classification ring diagram of metabolites of root samples at the super-class level. Different colors in the figure represent different classifications, and numbers in the figure represent the amounts of metabolites in each classification.

Both positive and negative ion modes were applied in the non-targeted UHPLC-MS/MS analysis of *E. nutans* ‘Aba’ root samples to maximize the detection of metabolites. After preprocessing the original data of 24 samples from 4 treatment groups (each treatment group has 6 biological repeats), 1,010 metabolites were identified, including 566 and 444 metabolites in the positive and negative ion modes, respectively. At the superclass level, these metabolites were divided into 10 categories, the top four categories were lipids and lipid molecules (14.26%), organic acids and derivatives (8.12%), phenylpropanoids and polyketides (7.03%) and organoheterocyclic compounds (6.73%) (Figure 3).

### Multivariate statistical analysis of metabolites

**Figure 4:**
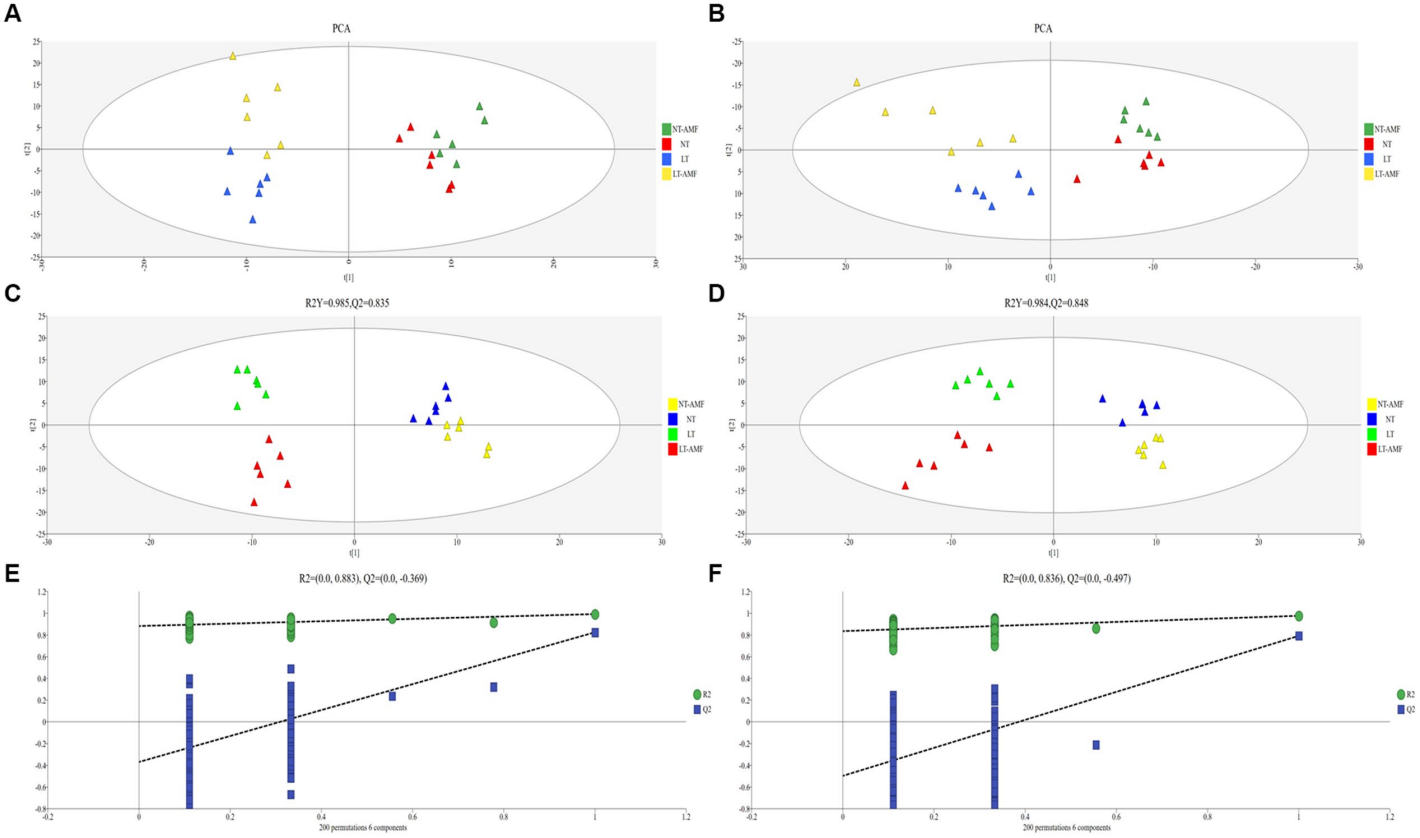
**(A)** PCA diagram in positive ion mode, **(B)** PCA diagram in negative ion mode, **(C)** PLS-DA analysis in positive ion mode, **(D)** PLS-DA analysis in negative ion mode, **(E)** permutation test in positive ion mode, and **(F)** permutation test in negative ion mode.

Principal component analysis (PCA) was applied to visualize the differences between the four treatment groups. According to the PCA diagrams of the positive and negative ion modes, six samples from the same group gather together, while there was a certain distance between samples from different groups (Figures 4A,B), indicating that the repeatability within the sample group was good. However, there were certain differences between the different groups. The samples of the normal temperature groups and the low temperature groups distributed on both sides of the Figures 4A,B, suggesting a significant difference of the metabolites between the normal temperature-treated groups (i.e., NT and NT-ANF) and the low temperature-treated groups (i.e., LT and LT-AMF). However, the samples of the inoculated and non-inoculated groups clustered, indicating that the metabolites of the inoculated and non-inoculated groups were similar.

Partial least squares discriminant analysis (PLS-DA) was performed on the metabolite accumulation level to investigate the reliability of metabonomics data. As shown in Figures 4C,D, in positive ion mode, the R2Y of the PLS-DA model was 0.985 and Q2 was 0.835; in negative ion mode, the R2Y of the PLS-DA model was 0.984 and Q2 was 0.848. The values of R2Y and Q2 in the positive and negative ion modes were close to 1, indicating that the PLS-DA model had good recognition and prediction ability. At the same time, the PLS-DA model was verified through random array tests (200 times). According to verified results, the R2 and Q2 values of random array tests were lower than the original values, and the intersection point of the blue regression line and the *Y*-axis was below zero (Figures 4E,F), indicating that the PLS-DA models did not have over-fitting, i.e., the model was reliable and applicable in analyzing the differences in metabolites between treatment groups.

### Analysis of differential metabolites

**Figure 5:**
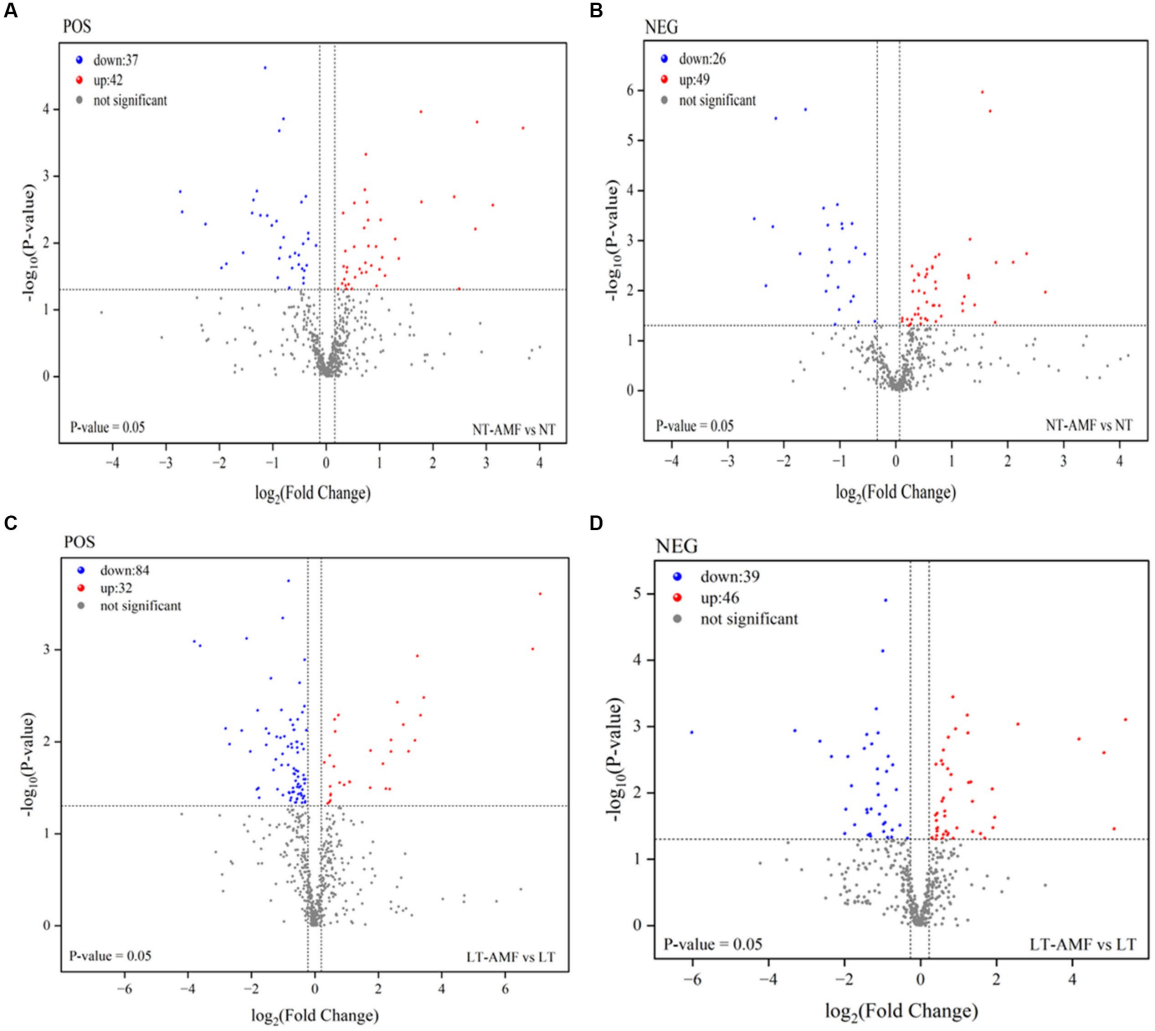
Volcano plots of different metabolites among sample groups. At room temperature, the differential metabolites between the inoculated and non-inoculated groups in the positive **(A)** and negative **(B)** ion modes. After low-temperature stress, the differential metabolites between inoculated and non-inoculated groups under positive **(C)** and negative **(D)** ion modes.

Differential metabolites (DAMs) were identified between the four treatment groups (*t*-test, *p* < 0.05, absolute log_2_FC >1, and VIP >1) and visualized by the volcano plots (Figure 5). Between the inoculated and the non-inoculated groups (i.e., NT vs. NT-AMF), 79 and 75 DAMs were identified in the positive and negative ion modes, respectively. Among them, 42 and 49 DAMs were up-regulated (red dots) in the positive and negative ion modes, respectively, and 37 and 26 DAMs were down-regulated (blue dots) in the positive and negative ion modes, respectively (Figures 5A,B). After 10 days of low temperature stress, 116 and 85 DAMs were identified between the inoculated and non-inoculated groups (i.e., LT vs. LT-AMF) in the positive and negative ion modes, respectively. As show in Figure 5, up-regulated metabolites (red dots) were 32 and 46 DAMs in the positive and negative ion modes, respectively, and 84 and 39 DAMs (blue dots) were down-regulated in the positive and negative ion modes, respectively (Figures 5C,D). At the superclass level, these DAMs included lipids and lipid molecules, benzenoids, organoheterocyclic compounds, organic oxygen compounds, phenylpropanoids and polyketones, nucleotides and analogues, and organonitrogen compounds. According to biological functions of metabolites, these differentially expressed metabolites can be divided into lipids, nuclear acids, antibiotics, peptides, steroids, vitamins and cofactors, and organic acids.

**Figure 6:**
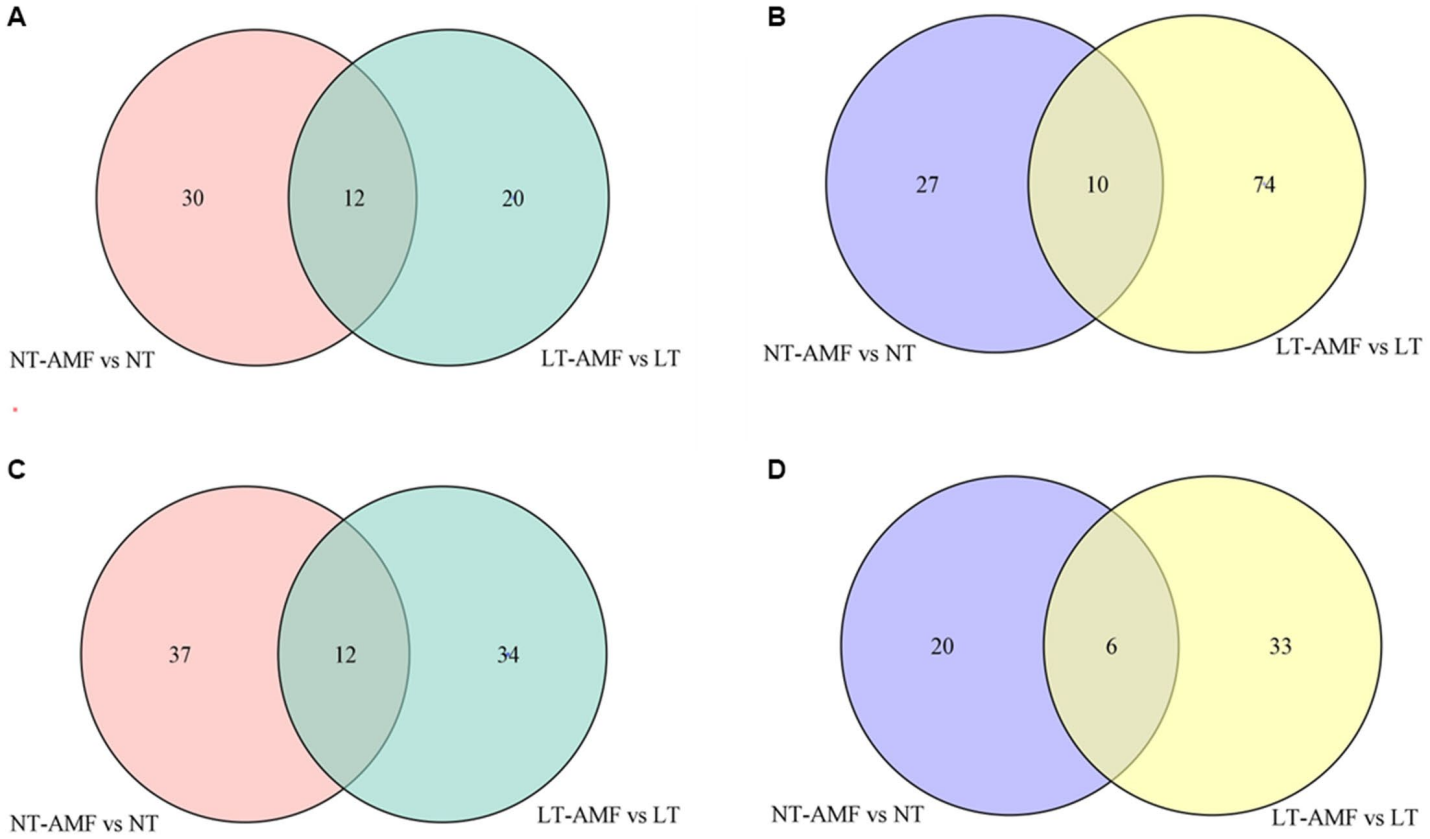
Venn diagrams of different metabolites between the inoculated and non-inoculated groups treated with normal or low temperature. **(A)** Common up-regulated metabolites in positive ion mode; **(B)** common down-regulated metabolites in positive ion mode; **(C)** common up-regulated metabolites in negative ion mode; **(D)** common down-regulated metabolites in negative ion mode.

**Figure 7:**
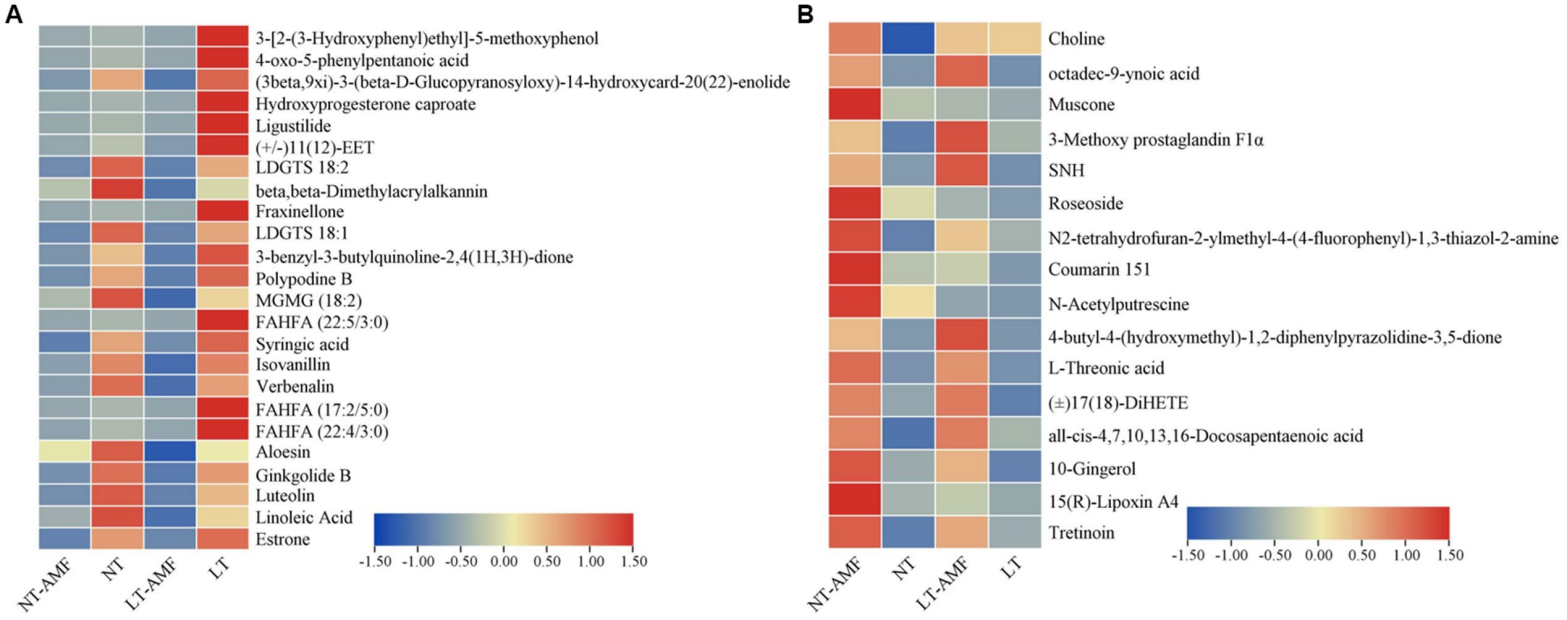
Cluster heatmaps of common differential metabolites between the inoculated and non-inoculated groups treated with normal or low temperature. **(A)** The down-regulated metabolites in inoculated groups, **(B)** the up-regulated metabolites in inoculated groups.

The Venn diagrams were applied to visualize the unique and common up-regulated and down-regulated metabolites among the four treatment groups (Figure 6). In the two groups (i.e., NT vs. NT-AMF), 12 differential metabolites were up-regulated in the positive and negative ion modes, respectively (24 in total). Meanwhile, the common down-regulated metabolites were 10 and 6 in the positive and negative ion modes, respectively. The cluster heatmaps showed the similarities and differences between these up-regulated and down-regulated metabolites (Figure 7). Except for individual metabolites, the number of up-regulated metabolites in the non-inoculated groups (i.e., NT and LT) was significantly higher than that in the inoculation groups (i.e., NT-AMF and LT-AMF). On the contrary, the number of down-regulated metabolites in the inoculated groups was significantly higher than that in the non-inoculated groups.

### KEGG enrichment analysis

**Figure 8:**
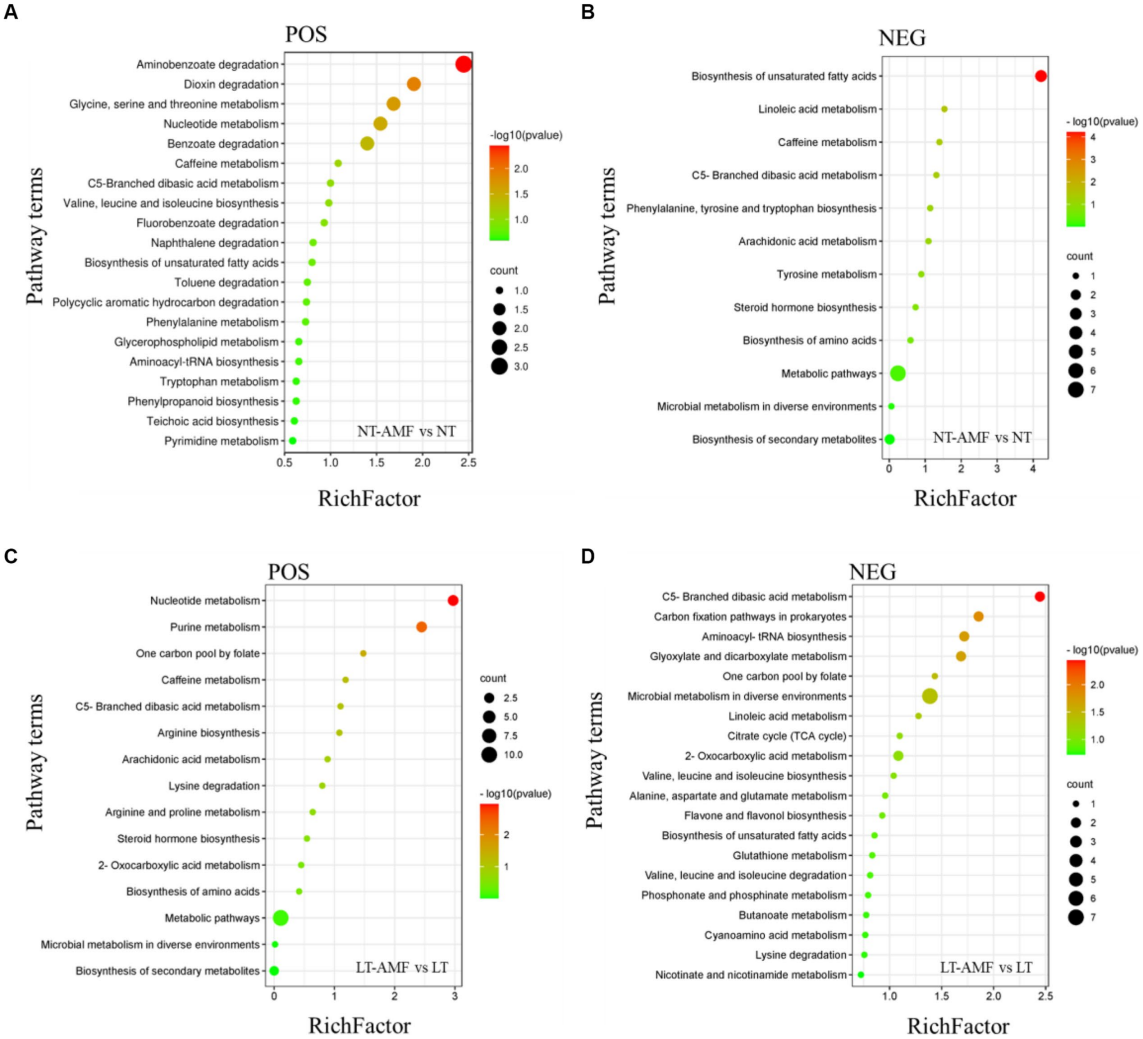
KEGG enrichment analysis of differential metabolites. **(A)** Metabolic pathways of DAMs enrichment in positive ion mode (NT-AMF vs. NT), **(B)** metabolic pathways of DAMs enrichment in negative ion mode (NT-AMF vs. NT), **(C)** metabolic pathways of DAMs enrichment in positive ion mode (LT-AMF vs. LT), **(D)** metabolic pathways of DAMs enrichment in negative ion mode (LT-AMF vs. LT).

KEGG database can link metabolites with specific metabolic pathways based on the differential accumulation of metabolites. Through KEGG enrichment analysis, 12–34 enrichment pathways were obtained in the positive and negative ion modes, and enrichment bubble diagrams with the first 12–20 enrichment values were shown (Figure 8).

In the comparison between NT and NT-AMF (Figures 8A,B), five metabolic pathways were significantly enriched in positive ion mode, including aminobenzoate degradation, dioxin degradation, glycine, serine and threonine metabolism, nucleotide metabolism, and benzoate degradation. Meanwhile, four metabolic pathways were significantly enriched in the negative ion mode, including biosynthesis of unsaturated fatty acids, linoleic acid metabolism, caffeine metabolism, and C5-blanched dibasic acid metabolism.

In the comparison between LT and LT-AMF (Figures 8C,D), three metabolic pathways were significantly enriched in positive ion mode, including nucleotide metabolism, purine metabolism, and one carbon pool by folate. Under the negative ion mode, 6 metabolic pathways were significantly enriched, including C5-branched dibasic acid metabolism, carbon fixation pathways in prokaryotes, aminoacyl tRNA biosynthesis, glyoxylate and dicarboxylate metabolism, one carbon pool by folate, and microbial metropolis in diverse environments.

In the comparison between NT vs. NT-AMF and LT vs. LT-AMF (Figure 8), there were three enriched metabolic pathways in the positive ion mode, including nucleotide metabolism, caffeine metabolism, and C5-branched dibasic acid metabolism. Among them, the only common metabolic pathway with significant enrichment was nucleotide metabolism. In the negative ion mode, there were four enriched metabolic pathways, including C5-branched basic acid metropolis, microbial metropolis in diffuse environments, linoletic acid metropolis, and biosynthesis of unsaturated fatty acids.

### Relationship between antioxidant enzyme activities and flavonoid or lipid metabolites

**Figure 9:**
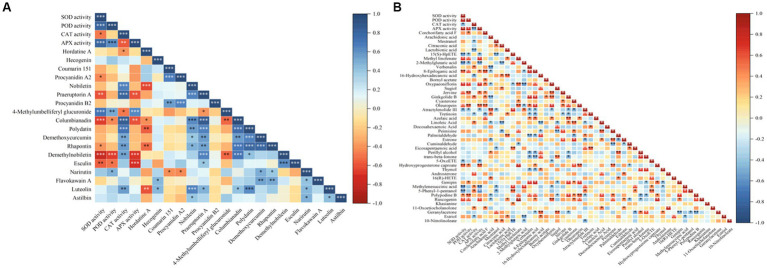
Correlation heatmaps between antioxidant enzyme activities and antioxidant related metabolites. **(A)** The correlation heatmap of antioxidant enzyme activities and flavonoid metabolites, **(B)** the correlation heatmap of antioxidant enzyme activities and lipids and lipid-like molecules.

Some studies showed that lipids and lipid molecules, and flavonoid metabolites play important roles in the defence responses of plants (Okazaki and Saito, 2014; Yang et al., 2018). In order to clarify the relationship between antioxidant enzyme activities and lipid or flavonoid metabolites, 33 flavonoid metabolites, 144 lipids and their derivatives were screened from all metabolites at first. Then, 18 flavonoid metabolites (DAMs) and 45 lipids and their derivatives (DAMs) were selected. Finally, the correlation analysis with SOD, POD, CAT and APX were carried out. The heatmaps showed the relationship between the activities of four antioxidant enzymes and the flavonoid metabolites (Figure 9A) and lipids and lipid molecules (Figure 9B).

Figure 9 showed a significantly or extremely significantly positive or negative correlation between the activities of the four antioxidant enzymes and some flavonoids or lipids. Among them, the activities of SOD, POD, and APX were significantly positive correlation with the level of 4-methyllumbelliferyl glucuronide (*p* < 0.01), and they were significantly negative correlation with the levels of esculin, demethylnobiletin and columbianadin. CAT activity was significantly positive correlation with the levels of luteolin, columbianadin, polydatin, demethoxycurcumin, rhapontin, demethylmobiletin, nobiletin, and praeruptorin A (*p* < 0.05), and negative correlation with the levels of hordatine A, and 4-methylumbelliferyl glucuronide (*p* < 0.05). At the same time, the activities of SOD, POD, and APX were significantly (*p* < 0.05) or extremely significantly (*p* < 0.01) positive correlation with the levels of mestranol, 8-epilogic acid, oxypaeoniflorin, jervine, oleuropein, hydroxyproterone capture, and ruscogenin, and were significantly (*p* < 0.05) or extremely significantly (*p* < 0.01) negative correlation with the levels of 15 (S)-HepETE, 2-methylglutamate acid and atractylolide III. CAT activity was significantly (*p* < 0.05) or extremely significantly (*p* < 0.01) positive correlation with the levels of verbenalin, ginkgolide B, linoleic acid, androsterone, methylenesuccinic acid, 5-phenyl-1-pentanol, and 11-oxoetocholinolone, and was extremely significantly (*p* < 0.01) negative correlation with the levels of mestranol, lactobionic acid, 16-hydroxyhexadecanoic acid, tretinoin, and sstriol. These results indicated that the relationship between antioxidant enzyme activities and flavonoid and lipid metabolites was related to the types and levels of metabolites.

## Discussion

Plants are inevitably affected by various environmental stresses in their whole life process. In order to respond to these disturbances, plants have evolved related resistance mechanisms, such as increasing the activities of antioxidant enzyme and changing the levels of metabolites (Ma et al., 2021; Malicka et al., 2021). In this study, our results showed that after 10 days of cold stress treatment, the antioxidant enzyme activities (except for CAT) of *E. nutans* roots significantly increased, which was consistent with previous studies (Sun et al., 2017; Yan et al., 2021). The enhancement of antioxidant enzyme activities in plants were mainly related to the increase of reactive oxygen species (ROS) caused by low temperature. However, the increasing rates in the inoculation groups were lower than those in the non-inoculation groups (Figure 2), which mainly attributed to the stress time. On the one side, short-term cold stress likely resulted in a rapid increase in antioxidant enzyme activities in the inoculated group, with a large amount of energy expenditure. On the other side, energy deficiency could reduce antioxidant enzyme activities with extended stress time (Lyu et al., 2022). It was also possible that the non-inoculated groups could only reduce oxidative damage by significantly increasing the activities of antioxidant enzyme to cope with cold stress. However, the inoculated groups may reduce the content of ROS in plants due to the presence of AMF, resulting in less increase of activities of antioxidant enzyme.

Changes in metabolites and their levels are considered to be the ultimate response of plants to environmental stresses (Ge et al., 2020). Plant metabolites can be divided into primary (such as carbohydrates, lipids and proteins, etc.) and secondary metabolites (phenols, flavonoids, alkaloids and polyamines, etc.), and the levels of metabolites are closely related to the plant itself and environmental factors (Aversano et al., 2017; Yang et al., 2018). Through metabolomic analysis, metabolites and their levels in plants can be comprehensively analyzed. In our study, through UHPLC-MS/MS analysis, 1,010 metabolites were detected in the roots of *E. nutans*, including lipids and lipid-like molecules, organic acids and derivatives, organoheterocyclic compounds, phenylpropane and polyketones, benzenoids, organic oxygen compounds, nucleosides & nucleotides and analogues (Figure 3). Among them, lipids and lipid-like molecules accounted for the highest proportion, indicating that lipids were the main components of the metabolites of Gramineae, which was similar to the result of Bernardo et al. (2019). Lipids are the main components of biofilms and provide energy for various physiological processes, plants can cope with low-temperature stress by changing lipid composition or level (Takahashi et al., 2013). The results of PCA, PLS-DA and 200 random permutations showed that the sequencing results were reliable and could be applied for subsequent analysis (Figure 4). There was a significant separation between the low- temperature and the room-temperature groups, but the inoculated and non-inoculated groups were similar, suggesting that the effect of low temperature on metabolites may be greater than that of the inoculation of AMF, the more direct influence of abiotic environmental factors on plants may be the main reason for this phenomenon. However, the effect of AMF on the metabolites of *E. nutans* roots should be addressed.

We applied three criteria to screen the differential metabolies (i.e., *t*-test for *p* < 0.05, log_2_FC >1, and VIP >1). A total of 154 (room temperature) and 201 (low temperature) differential metabolites were detected between the inoculation and non-inoculation groups, including 91 (room temperature) and 78 (low temperature) up-regulated metabolites, and 63 (room temperature) and 123 (low temperature) down-regulated metabolites (Figure 5). These results showed more differential metabolites in the low temperature groups than in the room temperature groups, but it appeared to be less than in the results of previous studies (Liu et al., 2021; Xie et al., 2022). The sampling time of roots of *E. nutans* in the late stage of AMF development was probably the main reason for fewer DAMs.

The KEGG enrichment analysis shown that the most of these differential metabolites were enriched in metabolism-related pathways. In comparing inoculated and non-inoculated plants under room and low temperatures, differential metabolites were significantly enriched in nucleotide metabolism and C5-branched dibasic acid metabolism (Figure 8). In conclusion, inoculation of the AMF may regulate plant cold resistance by affecting the metabolism in plants. In addition, 24 up-regulated metabolites and 16 down-regulated metabolites were screened between the inoculated and non-inoculated groups under room and low temperature (Figure 7). These metabolites were mainly lipids and flavonoids, which was in consistent with previous studies (Chen et al., 2013; Zhou et al., 2018). The reasonable explanation was that lipids and flavonoids played important roles in enhancing plant stress resistance and removing ROS effectively. The increase of their contents may be related to the accumulation of ROS and synthesis of some certain compounds caused by low temperature (Mahajan and Tuteja, 2005; Agati et al., 2011; Landi et al., 2015). These results indicated that AMF inoculation might affect the cold resistance of plants by affecting the levels of metabolites, such as lipids and flavonoids, related to levels of plant antioxidants.

Many studies have shown that lipids and their derivatives play key roles in the defence response of plants, affecting the resistance mechanisms related to plant-microbe interactions (Feng et al., 2020, 2022), and flavonoids are one of the main secondary metabolites in plants, with antioxidant properties. Increase in flavonoid levels helps plants strengthen their resistance to abiotic stresses, such as cold stress (Ren et al., 2019; Shah and Smith, 2020). In this study, 33 flavonoid metabolites, including flavonoids, isoflavones, and 2-arylbenzofuran flavonoids, were screened in all samples, and 18 of them showed differential changes. 144 lipids and their derivatives were screened, and 45 of them showed differential changes. According to the results of correlation analysis between these 18 flavonoids metabolites and 45 lipids and their derivatives and the activities of SOD, POD, CAT, and APX, there were significantly or extremely significantly positive or negative correlations between the activities of the four antioxidant enzymes and some metabolites in flavonoids and lipids (Figure 9). The reason is that antioxidant enzymes, lipids, and flavonoids are important factors in removing ROS and alleviating oxidative damage, and there is a certain synergistic effect between them (Janda et al., 2003). These results indicate that the activities of antioxidant enzyme in the root of *E. nutans* were correlated with the levels of flavonoids and lipid metabolites, and the degree of correlation was related to the types and levels of the metabolites.

In conclusion, the regulation of AMF on cold-resistance of *E. nutans* may be realized by affecting the metabolic activities of some organic acids, such as nucleotide metabolism, etc., as well as the levels of some flavonoids and lipid metabolites related to antioxidant effects. However, these results may be insufficient to reveal the mechanism under different growth stages, stress intensities and duration. Therefore, time-course metabolomics and microscopy of AMF development may be required for further insights into the dynamics of mycorrhizal effect at the metabolic level.

## Conclusion

Inoculation of *R. irregularis* at room temperature had no significant effect on the activities of antioxidant enzyme in the roots of *E. nutans* ‘Aba’, but significantly changed the levels of some lipids and other metabolites. However, the activities of antioxidant enzyme and levels of some metabolites were significantly changed under cold stress. Meanwhile, most of these metabolites were enriched in the pathways related to plant metabolism, and the activities of antioxidant enzyme were closely related to the levels of some metabolites, such as flavonoids and lipids. These results can provide some basis for studying the molecular mechanism of AMF regulating cold-resistance of Gramineae grasses.

## Data availability statement

The datasets presented in this study can be found in online repositories. The names of the repository/repositories and accession number(s) can be found below: Metabolights accession number MTBLS7231.

## Author contributions

HaZ, HQ, XZ, GL, JuW, YF, HuZ, and CW conceived and designed the experiments. HaZ, HQ, XZ, JL, KZ, and JiW performed the experiments. HaZ, HQ, GL, and CW analyzed the data. HaZ and HQ drew pictures and wrote the paper. All authors contributed to the article and approved the submitted version.

## Funding

This study was financially supported by the Basic Research Project of Qinghai Provincial Science and Technology Department (2021-ZJ-915).

## Conflict of interest

The authors declare that the research was conducted in the absence of any commercial or financial relationships that could be construed as a potential conflict of interest.

The reviewer JU declared a past co-authorship with the author HuZ to the handling editor.

## Publisher’s note

All claims expressed in this article are solely those of the authors and do not necessarily represent those of their affiliated organizations, or those of the publisher, the editors and the reviewers. Any product that may be evaluated in this article, or claim that may be made by its manufacturer, is not guaranteed or endorsed by the publisher.

## Footnotes

^1^ http://www.bioinformatics.com.cn/
